# Supplementary material for: Theoretical open-loop model of respiratory mechanics in the extremely preterm infant
Source: PLoS One. 2018 Jun 14;13(6):e0198425. doi: 10.1371/journal.pone.0198425 (PMC6002107; doi:10.1371/journal.pone.0198425)
Supplement: S1 Appendix — The inherent stability of the model was analyzed under constant non-oscillatory muscle pressure by examining the eigenvalues of the Jacobian at the nominal parameter set and varying parameters by integer multiples. (PDF) [file pone.0198425.s001.pdf]

S1 Appendix

We analyze the inherent stability of the model under constant non-oscillatory muscle pressure by examining the eigenvalues of the Jacobian at the nominal parameter set and varying parameters by multiples of 2 and 10. To obtain steady-states, P_{mus} is set at a constant called $P_{mus,C}$ and the system of ODE's is set to equal 0:

$$\begin{aligned}
0 &= \frac{1}{I_u}\left(P_{ao} - P_u - R_u\dot{V}\right) \\
0 &= \dot{V} - \dot{V}_A \\
0 &= \frac{\dot{V}_A}{C_A} \\
0 &= \frac{\dot{V}_A - (P_{ve}/R_{ve})}{C_{ve}}
\end{aligned}$$

It follows that $\dot{V} = \dot{V}_C = \dot{V}_A = 0$ and $P_u = P_{ve} = P_c = P_A = 0$. Given the relations $P_{el} = f(V_A)$, $P_{tm} = f(V_c)$, and $P_{cw} = f(V_{cw})$, we have 3 equations with 6 unknowns $(V_A, V_c, V_{cw}, P_{el}, P_{tm}, P_{cw})$. Three additional equations come from incorporating loop summations such that $P_{el}(V_A) = P_{tm}(V_c)$, $P_{tm} = -(P_{cw} + P_{mus,C})$, and $V = V_{cw} = V_A + V_c$. Noting that P_{el} is defined implicitly by Eq. (10) and using the compliance curve functions

$$\begin{aligned}
P_{cw} &= c_w + d_w \ln\left(e^{(V_A + V_c - a_w)/b_w} - 1\right) \\
P_{tm} &= c_c - d_c \ln\left(\frac{V_{c,max}}{V_c} - 1\right)
\end{aligned}$$

we obtain two equations

$$\begin{aligned}
0 &= P_{cw}(V_A(P_{el}), V_c) + P_{mus,C} + P_{tm}(V_c) \\
0 &= P_{cw}(V_A(P_{el}), V_c) + P_{mus,C} + P_{el}
\end{aligned}$$

that are solved numerically for two unknowns P_{el}, V_c for each modified parameter set using an iterative algorithm. P_{el} From these steady-state values we can calculate the remaining state variables.

In order to linearize the system and determine asympototic behavior we rewrite the system in terms of the state variables $\dot{V}, V_c, P_{el}, P_{ve}$ using previously described relationships:

$$\begin{aligned}
\ddot{V} &= \frac{1}{I_u}\left(P_{ao} - R_c(V_c)\dot{V} - P_{tm}(V_c) - P_{cw}(V_A(P_{el}) + V_c) - P_{mus} - R_u(\dot{V})\dot{V}\right) \\
\dot{V}_c &= \dot{V} - \frac{P_{tm}(V_c) - P_{el} - P_{ve}}{R_s(V_A(P_{el}))} \\
\dot{P}_{el} &= \frac{1}{C_A(P_{el})}\left[\frac{P_{tm}(V_c) - P_{el} - P_{ve}}{R_s(V_A(P_{el}))}\right] \\
\dot{P}_{ve} &= \frac{1}{C_{ve}}\left[\frac{P_{tm}(V_c) - P_{el} - P_{ve}}{R_s(V_A(P_{el}))} - \frac{P_{ve}}{R_{ve}}\right]
\end{aligned}$$

where the quantity $\frac{P_{tm}(V_c)-P_{el}-P_{ve}}{R_s(V_A(P_{el}))} = \dot{V}_A$. The Jacobian

$$J = \begin{bmatrix} \frac{\partial \ddot{V}}{\partial \dot{V}} & \frac{\partial \ddot{V}}{\partial V_c} & \frac{\partial \ddot{V}}{\partial P_{el}} & \frac{\partial \ddot{V}}{\partial P_{ve}} \\ \frac{\partial \dot{V}_c}{\partial \dot{V}} & \frac{\partial \dot{V}_c}{\partial V_c} & \frac{\partial \dot{V}_c}{\partial P_{el}} & \frac{\partial \dot{V}_c}{\partial P_{ve}} \\ \frac{\partial \dot{P}_{el}}{\partial \dot{V}} & \frac{\partial \dot{P}_{el}}{\partial V_c} & \frac{\partial \dot{P}_{el}}{\partial P_{el}} & \frac{\partial \dot{P}_{el}}{\partial P_{ve}} \\ \frac{\partial \dot{P}_{ve}}{\partial \dot{V}} & \frac{\partial \dot{P}_{ve}}{\partial V_c} & \frac{\partial \dot{P}_{ve}}{\partial P_{el}} & \frac{\partial \dot{P}_{ve}}{\partial P_{ve}} \end{bmatrix}$$

was found using symbolic computation then used numerically to calculate eigenvalues. All parameter variations gave stable solutions except c_c, d_c, and $V_{c,max}$. However, varying these parameters by 2 and 10 is not actually physiological and the instability comes from these modifications making the parameter values inconsistent with the rest of the system. The parameters c_c and d_c must be equal with our calculations, and since c_c is a normal operating pressure, it must stay within a narrow range. Likewise, $V_{c,max}$ is equivalent to dead space so not only must it also be within a narrow range but if it varies too much with respect to other parameters and states then the equations will be inconsistent. It is therefore reasonable to state that the steady-state of this model system is inherently stable for the physiological parameter ranges used here.
