# Supplementary material for: Theoretical open-loop model of respiratory mechanics in the extremely preterm infant
Source: PLoS One. 2018 Jun 14;13(6):e0198425. doi: 10.1371/journal.pone.0198425 (PMC6002107; doi:10.1371/journal.pone.0198425)
Supplement: S1 Code — Attached MATLAB code runs Simulation 3 (high Cw, normal Ru) with constant frequency for 6 periods. (PDF) [file pone.0198425.s002.pdf]

The MATLAB code shown here is set up for Simulation 3 (high C_w, normal R_u) with constant frequency, run for 6 periods.. The script `compliance_curves.m` generates a .mat file to be used for plotting in `VentilationModel_solver.m`. The script `driver.m` runs the remainder of the code.

compliance_curves.m

```
clear all

TLC=.001*63;
RV=.001*23;
VC=(TLC-RV);

alpha_cw=0.25;%
volume0Pres=(alpha_cw*VC+RV);
aw=RV;
cw=0;
dw=2*1.2*.2;
bw=alpha_cw*VC/(log(1+exp(-cw/dw)));

Pel=[-20:.01:35]';
Pcw=[-20:.01:35]';
Phalf =.1;
Ptau =.5;
beta=.01;
gamma =1;
k=0.07;

alpha=((1+exp(Phalf/Ptau))*beta-gamma)/exp(Phalf/Ptau);
Frec=alpha+(gamma-alpha)./(1+exp(-(Pel-Phalf)/Ptau));
Vel=VC*(1-exp(-k*Pel));
VA=Frec.*Vel+RV;
Vcw=aw+bw*log(1+exp((Pcw-cw)/dw));

itemp_start=find(Pcw==-15);
iVcw_start=find(abs(Vcw-Vcw(itemp_start))<0.0005, 1, 'last' ); %volume Vcw at
that index
iVA_start=find(abs(VA-Vcw(itemp_start))<.0005, 1, 'last' ); %volume VA at the
same index

itemp_end=find(abs(Vcw-TLC)<0.02,1, 'last');
iVcw_end=find(abs(Vcw-Vcw(itemp_end))<0.01, 1, 'last' );
iVA_end=find(abs(VA-Vcw(itemp_end))<1, 1, 'last' );

pcwt=Pcw(iVcw_start:iVcw_end);
vcwt=Vcw(iVcw_start:iVcw_end);
pelt=Pel(iVA_start:iVA_end);
vat=VA(iVA_start:iVA_end);

VolN=linspace(min(vat),max(vat),3000);
PelN=interp1(vat,pelt,VolN);
PcwN=interp1(vcwt,pcwt,VolN);
Ptot=PelN+PcwN;
```

```
index=find(abs(Ptot)<0.01,1,'last');
FRC=VolN(index);
P_FRC=PelN(index);

Pel_range=Pel;Vcw_range=Vcw;VA_range=VA;
save PVcurves.mat Pel_range Vcw_range VA_range Ptot VolN FRC P_FRC

figure
plot(Pcw,Vcw,'b',Pel(2001:end),VA(2001:end),'r',Ptot,VolN,'m');%Pel,Vel+RV,'g
',
hold on
plot(PcwN(index),FRC,'k*',PelN(index),FRC,'k*')
axis([-10 35 RV TLC])
line([0 0],[0 TLC],'Color',[.7 .7 .7])
line([-10 35],[RV RV],'Color',[.4 .4 .4],'Linestyle','--')
line([-10 35],[FRC FRC],'Color',[.4 .4 .4],'Linestyle',':')
xlabel('Pressure')
ylabel('Volume, ml')
legend('Chest wall','Lung','Total (chest plus lung)','FRC','Location','best')
```

driver.m

```
global dwMult RuMult

%%% Toggle for Cw and Ru
% stateCw='low';
stateCw='high';
stateRu='norm';
% stateRu='high';

if strcmp(stateCw,'low')==1
    dwMult=1;
else
    dwMult=0.2;
end
if strcmp(stateRu,'norm')==1
    RuMult=1;
else
    RuMult=10;
end


load_VentilationModel_pars
NP=6; %Number of periods
VentilationModel_solver(NP)
```

load_VentilationModel_pars.m

```
close all

global Rum Ku Kc Vcmax Rsd Ks Rsm Vstar0 RV TLC0
global  Cve Rve FRC I cc dc VC  volume0Pres
```

```
global RR f T Amus aw bw cw dw Phalf Ptau k
global beta gamma alpha
global dwMult RuMult


Phalf =.1;
Ptau = .5;
beta=0.01;
gamma = 1;
alpha=((1+exp(Phalf/Ptau))*beta-gamma)/exp(Phalf/Ptau);
k=0.07;

FRC   = 0.02489;%0.0281;
RV    = .001*23;
TLC0   = .001*63;
VC  = TLC0-RV;
alpha_cw=0.25;%
volume0Pres = alpha_cw*VC+RV;
RR      = 60;
f       = RR/60;
T       = 1/f;

aw=RV;
bw=(1/log(2))*(volume0Pres-RV);
cw=0;
dw=2*1.2*dwMult;

if dwMult==1 %low Cw
    if RuMult==1  %normal Ru
        halfAmus=1.39;
    else
        halfAmus=1.9;
    end
else   % high Cw
    if RuMult==1  %normal Ru
        halfAmus=0.925;
    else
        halfAmus=1.6;
    end
end
Amus   = 2*halfAmus;

cc    = 4.4;
dc    = 4.4;
Rum   = 20;
Ku    = 60;
I     = 0.33;
Kc    = .1;
Vcmax = 0.0025;
Rsm    = 12;
Rsd    = 20;
Ks    = -15;
Vstar0 = TLC0;
Cve   = 0.005;
Rve   = 20;
```

VentilationModel_solver.m

```
function VentilationModel_solver(NP)

global Rum Ku Kc Vcmax  Ks  Vstar0 RV TLC
global  Amus f  T FRC Rsd Rsm %NP
global cc dc TLC0  Phalf Ptau k
global beta gamma alpha VC aw bw cw dw fracOpen tcpap RuMult
global i

tstep=0.01;
NumAlv=10000;
Ns=NumAlv;

f=1/T;
tprev=0;
tnext=tprev+T;
periods=[tprev tnext];
tspan = [tprev:tstep:tnext];
ppp=length(tspan);

Vdot0=0;
Pel0=0.954;  %%% Pel0 for low Cw is 2.015;
Vc0 = 0.0001;
Pve0 = 0;

Init=[Vdot0 Pel0 Vc0 Pve0];
ts=0;
times=0;
sols=Init;

fsave=f;
Vstarsave=Vstar0;
gammasave=gamma;
alphasave=alpha;
Phalfsave=Phalf;
Ptausave=Ptau;
Pmussave=0;
VTsave=0.005;
VAminsave=[];
Cdynsave=[];
Cwdynsave=[];
fracOpensave=[];

TLC=TLC0;
TLCsave=TLC;
Phalf0=Phalf;
gamma0=gamma;
Pmult=1;
Ptau0=Ptau;
fracAtel=0;
tcpap=1e10;
count=0;
ttemp=0;
Amus0=Amus;
```

```
options = [];

for i=1:NP
    try
        [time,sol]=ode15s(@VentilationModel,tspan,Init,options,T,tprev);
%,options
        times=[times;time(2:end)];
        sols=[sols; sol(2:end,:)];
        Init=[sol(end,1:4)];
        Peltemp=sol(2:end,2);
        maxPel=max(Peltemp);
        minPel=min(Peltemp);
        endPel=Peltemp(end);
        alpha=((1+exp(Phalf/Ptau))*beta-gamma)/exp(Phalf/Ptau);
        Frectemp=alpha+(gamma-alpha)./(1+exp(-(Peltemp-Phalf)/Ptau));
        fracOpen=max(Frectemp);
        minFrec=min(Frectemp);
        endFrec=Frectemp(end);
        Veltemp=VC*(1-exp(-k*Peltemp));
        VAtemp=Frectemp.*Veltemp+RV;
        VAmax=max(VAtemp);
        VAmin=min(VAtemp);
        Cdyn=(VAmax-VAmin)/(maxPel-minPel);
        Vcwtemp=VAtemp+sol(2:end,3);
        Vcwmax=max(Vcwtemp);
        Vcwmin=min(Vcwtemp);
        maxPcw=cw+dw*log(exp((Vcwmax-aw)/bw)-1);
        minPcw=cw+dw*log(exp((Vcwmin-aw)/bw)-1);
        Cwdyn=(Vcwmax-Vcwmin)/(maxPcw-minPcw);
        VT=VAmax-VAmin;
        VE=VT*f*60;
        VAminsave=[VAminsave;VAmin];
        Cdynsave=[Cdynsave;Cdyn];
        Cwdynsave=[Cwdynsave;Cwdyn];
        fsave=[fsave;f];
        VTsave=[VTsave;VT];
        fracOpensave=[fracOpensave;fracOpen];
%%% Toggle for muscle pressure driver function
%         for j=1:length(tspan)-1
%             Pmussave = [Pmussave;VarFreqCosPmus(tspan(j)-tprev,T)];
%variable frequency
%         end
        Pmustemp=(Amus*cos(2*pi*f*(tspan-tprev)) + -Amus)'-0; %constant
frequency
        Pmussave=[Pmussave; Pmustemp(2:end)];
%       Pmussave=[Pmussave;ones(ppp-1,1)*-Amus]; %constant
        fracAtel=1-fracOpen;
        Ne=fracOpen*NumAlv;
        Nc=Ns-Ne;
        if mod(i,1)==0

disp([i,VAmax*1000,VAmin*1000,f,VT*1000,VE*1000,Cdyn*1000,Cwdyn*1000,fracOpen
,minPel,Ns,Ne])
        end
        if i==59
```

```
            fprintf('.')
            VEset=VE;
        end
        if i>=60
%%% Toggle for calculating variable frequency
%              VTmean=mean(VTsave(end-60:end));
%              f=VEset/(VTmean*60);
            EELV=VAmin;
            EILV=VAmax;
            EELVsac=EELV/NumAlv;
            EILVsac=EILV/NumAlv;
            Vpredsac=(EILVsac-fracAtel*EELVsac)/(1-fracAtel);
            PmultT=(Vpredsac/EILVsac)^(1/3);
            Pmult=Pmult*PmultT;
            Ptau=Ptau0*Pmult;
            Phalf=Phalf0*Pmult;
%%% Toggle for calculating variable gamma
            gamma=gamma0*1;%(1-fracAtel*.1);
            Ns=Ne;
        else
        end
        alpha=((1+exp(Phalf/Ptau))*beta-gamma)/exp(Phalf/Ptau);
        Vstarsave=[Vstarsave; ones(ppp-1,1)*Vstar0*gamma];
        gammasave=[gammasave; ones(ppp-1,1)*gamma];
        alphasave=[alphasave; ones(ppp-1,1)*alpha];
        Phalfsave=[Phalfsave; ones(ppp-1,1)*Phalf];
        Ptausave=[Ptausave; ones(ppp-1,1)*Ptau];
        T=1/f;
        tprev = tnext;
        tnext=tprev+T;
        tspan = [tprev:tstep:tnext];
        ppp=length(tspan);
        pend=periods(end)+T;
        periods=[periods pend];
        if Ns < NumAlv/10

disp([i,VAmax*1000,VAmin*1000,f,VT*1000,VE*1000,Cdyn*1000,Cwdyn*1000,fracOpen
,minPel,Ns,Ne])
            break
        end
    catch

disp([i,VAmax*1000,VAmin*1000,f,VT*1000,VE*1000,Cdyn*1000,Cwdyn*1000,fracOpen
,minPel,Ns,Ne])
        time=[]; sol=[];
        break
    end
%%% Initiating variable levels of CPAP
    if fracOpen <= 0.97 && times(end) > 500 %0.95, 0.90
        ttemp=times(end);
        count=count+1;
    end
    if ttemp > 500 && count<=1
        tcpap=ttemp;
        ttemp=0;
    end
end
```

```
disp(['disp([i,VAmax*1000,VAmin*1000,f,VT*1000,VE*1000,Cdyn*1000,Cwdyn*1000,f
racOpen,minPel,Ns,Ne])'])
disp([times(end)]);

t=times;
Vdot=sols(:,1);  Pel=sols(:,2);  Vc=sols(:,3); Pve=sols(:,4);

Frec=alphasave+(gammasave-alphasave)./(1+exp(-(Pel-Phalfsave)./Ptausave));
Vel=VC*(1-exp(-k*Pel));
VA=Frec.*Vel+RV;

Vcw=VA+Vc;

Pcw=cw+dw*log(exp((Vcw-aw)/bw)-1);
Ptm = cc-dc*log(Vcmax./(Vc-0)-1);

Pldyn = Pel+Pve;
Ppl=Pcw+Pmussave;
PA=Pldyn+Ppl;
Pc=Ptm+Ppl;

figure
plot(t,Vc*1000,'.',t,VA*1000,'.',t,Vdot*1000,'.',t,Vcw*1000,'.')
title('Volumes')
legend('Vc','VA','Vdot','Vcw')

Rc =  Kc*(Vcmax./Vc).^2;
for k=1:length(Vdot)
        if Vdot(k)<0
            Ru(k) = (Rum+ Ku*abs(Vdot(k)))*RuMult;%
        else
    Ru(k) = Rum+ Ku*abs(Vdot(k));
        end
end
Ru=Ru';
Rs =  Rsd*exp(Ks*(VA-RV)./(Vstarsave-RV))+Rsm;
Pu=Vdot.*Rc+Pc;
Rtot=Rc+Ru+Rs;

load PVcurves.mat

start=101;
figure
plot(Pel_range,Vcw_range*1000,'b',Pel_range(2001:end),VA_range(2001:end)*1000
,'r',Ptot(start:end),VolN(start:end)*1000,'m');
%,Pldyn(start:end)+Pcw(start:end),VA(start:end),'.'
hold on
plot(Pldyn(start:end),VA(start:end)*1000,'k','LineWidth',2)%'Color',[.6 .6
.6],
plot(P_FRC,FRC*1000,-P_FRC,FRC*1000,'Color',[.4 .4 .4],'Linestyle',':')
title('V'); legend('Chest wall','Lung','Respiratory','Tidal loop, normal
R_u','Tidal loop, increased R_u,','FRC','Location','Southeast');%,,'Ptot v.
VA',
xlabel('Pressure')
```

```
ylabel('Volume, ml')
axis([-5 35 RV*1000 60])%TLC0*1000
line([0 0],[0 60],'Color',[.7 .7 .7])
line([-5 35],[FRC*1000 FRC*1000],'Color',[.4 .4 .4],'Linestyle',':')

figure
subplot(511)
plot(t,Pmussave); ylabel('P_{mus} (cmH_2O)'); %xlabel('time (s)')
subplot(512)
plot(t,Ppl); ylabel('P_{pl} (cmH_2O)'); %xlabel('time (s)')
subplot(513)
plot(t,PA); ylabel('P_A (cmH_2O)'); %xlabel('time (s)')
subplot(514)
plot(t,VA*1000); ylabel('V_A (ml)'); %xlabel('time (s)')
subplot(515)
plot(t,-Vdot*1000); ylabel('Air flow (ml/s)'); xlabel('time (s)')
```

VentilationModel.m

```
function [dpdt] =VentilationModel(t,p,T,tprev)

global Rum Ku Kc Vcmax Ks Vstar0 RV
global Amus f Cve Rve I Rsd Rsm
global cc dc TLC0 Phalf Ptau k aw bw cw dw beta gamma alpha VC tcpap
global RuMult

Vdot = p(1);
Pel = p(2);
Vc = p(3);
Pve = p(4);

%%% Toggle for muscle pressure drive function
% Pmus=VarFreqCosPmus(t-tprev,1/f);
Pmus = (Amus*cos(2*pi*f*(t-tprev)) + -Amus)-0;
% Pmus = -Amus;

TLC=TLC0;
alpha=((1+exp(Phalf/Ptau))*beta-gamma)/exp(Phalf/Ptau);
Frec=alpha+(gamma-alpha)./(1+exp(-(Pel-Phalf)/Ptau));
Vel=VC*(1-exp(-k*Pel));
VA=Frec*Vel+RV;

Vcw=VA+Vc;

CA = VC*k*exp(-Pel*k)*((gamma + exp(-Phalf/Ptau)*(gamma -
beta*(exp(Phalf/Ptau) + 1)))/(exp(-(Pel - Phalf)/Ptau) + 1) -...
    exp(-Phalf/Ptau)*(gamma - beta*(exp(Phalf/Ptau) + 1))) +...
    (exp(-(Pel - Phalf)/Ptau)*(gamma + exp(-Phalf/Ptau)*(gamma -
beta*(exp(Phalf/Ptau) + 1)))*(VC - VC*exp(-Pel*k)))/(Ptau*(exp(-(Pel -
Phalf)/Ptau) + 1)^2);

Pcw=cw+dw*log(exp((Vcw-aw)/bw)-1);
Ptm = cc-dc*log(Vcmax./(Vc-0)-1);
```

```
Pldyn=Pel+Pve;
Ppl=Pcw+Pmus;
PA=Pldyn+Ppl;
Pc=Ptm+Ppl;
Vstar=Vstar0*gamma;
Rc =  Kc*(Vcmax/Vc)^2;
Rs =  Rsd*exp(Ks*(VA-RV)/(Vstar-RV))+Rsm;
if Vdot<0
    Ru = (Rum+ Ku*abs(Vdot))*RuMult;%
else
    Ru = Rum+ Ku*abs(Vdot);
end
Ru=Ru;

Pu=Vdot*Rc+Pc;
VAdot=(Pc-PA)/Rs;
Vcdot=Vdot-VAdot;

%%% Toggle for initializing CPAP
% if tprev>tcpap
%     Pao=5;%5;
% else
Pao=0;
% end

dpdt =   [(Pao-Pu-Ru*Vdot)/I;               %Vdot
    (VAdot)/CA; ...                 %Pel
    Vcdot;                          %Vc
    (VAdot-Pve/Rve)/Cve];           %Pve
```
